# Examining the association between exposome score for schizophrenia and cognition in schizophrenia, siblings, and healthy controls: Findings from the EUGEI study

**DOI:** 10.1192/j.eurpsy.2023.957

**Published:** 2023-07-19

**Authors:** L. Fusar-Poli, E.-G.-W. 6. Investigators, G. Investigators, J. van Os, B. P. Rutten, S. Guloksuz

**Affiliations:** 1 Department of Brain and Behavioral Sciences, University of Pavia, Pavia, Italy; 2 Department of Psychiatry and Neuropsychology, School for Mental Health and Neuroscience, Maastricht University, Maastricht; 3 UMC Utrecht Brain Centre, University Medical Centre Utrecht, Utrecht University, Utrecht, Netherlands; 4 Department of Psychosis Studies, Institute of Psychiatry, Psychology & Neuroscience, King’s College London, London, United Kingdom; 5 Department of Psychiatry, Yale University School of Medicine, New Haven, United States

## Abstract

**Introduction:**

Schizophrenia spectrum disorders (SSD) are frequently associated with disturbances in both neurocognition and social cognition. The patoetiology of SSD derives from a complex interaction between genes and environment. Exposome score for schizophrenia (ES-SCZ) is a cumulative environmental exposure score for schizophrenia which have shown potential utility in risk stratification and outcome prediction.

**Objectives:**

To investigate whether ES-SCZ is associated with cognition in patients with SSD, unaffected siblings, and healthy controls.

**Methods:**

The present cross-sectional study included 1141 patients with SSD, 1332 unaffected siblings, and 1495 healthy controls, recruited in the Netherlands, Spain, Serbia, and Turkey. The Wechsler Adult Intelligence Scale (WAIS) was used to evaluate neurocognition, while the Degraded Facial Emotion Recognition (DFAR) task was used to assess social cognition. ES-SCZ was calculated based on our previously validated method. Associations between ES-SCZ and cognitive domains were analyzed by applying regression models in each group (patients, siblings, and controls), adjusted by age, sex, and country.

**Results:**

According to our preliminary analyses, no significant associations were found between ES-SCZ and cognition in patients with SSD. ES-SCZ was negatively associated with WAIS in unaffected siblings (B=−0.40, p=0.03) and controls (B=-0.63, p=0.004) and positively associated with DFAR in siblings (B=0.83, p=0.004). No significant association between ES-SCZ and DFAR was found in healthy controls.

**Conclusions:**

Our findings show that neurocognition and social cognition are oppositely associated with ES-SCZ. Longitudinal studies may clarify whether there is a cause-effect relationship between ES-SCZ and cognition. Further research should investigate whether ES-SCZ interacts with molecular genetic risk for schizophrenia to improve clinical chcracterization and outcome prediction in people with SSD.

**Disclosure of Interest:**

None Declared

